# Stakeholder consultation to facilitate implementation of interventions for prevention and promotion in mental health in Europe: introducing the design of the ICare Stakeholder Survey

**DOI:** 10.1093/eurpub/ckab045

**Published:** 2021-07-07

**Authors:** Martina Nitsch, Karin Waldherr, Michael Zeiler, Lisa Klesges, Corinna Jacobi

**Affiliations:** 1 Ferdinand Porsche FernFH—Distance-Learning University of Applied Sciences, Vienna, Austria; 2 Department for Child and Adolescent Psychiatry, Medical University of Vienna, Vienna, Austria; 3 School of Public Health, University of Memphis, Memphis, TN, USA; 4 Institut für Klinische Psychologie und Psychotherapie, Technische Universität Dresden, Dresden, Germany

## Abstract

**Background:**

Online interventions to prevent mental health problems have proven to be effective. However, knowledge about their implementation in real-world practice as well as for dissemination to the target groups in different settings is scarce. The goal of the ‘ICare’ network is to establish a comprehensive model of eMental-health service delivery in and across different European countries. Since implementation and dissemination are influenced by many contextual factors, in the first phase of ICare a stakeholder survey was conducted. The survey aim was to explore stakeholders’ experiences, needs and attitudes regarding Internet-based prevention of mental health problems and hindering and fostering factors for implementation and dissemination. This article is part of a supplement and describes the design of the stakeholder survey. Survey results are published in separate articles in the same supplement.

**Methods:**

Based on a literature review and the individual characteristics of the ICare interventions, stakeholder groups were identified in different settings across six European countries. The RE-AIM framework guided the development of the research questions and survey instruments. A concurrent mixed methods design was applied comprising focus groups with the intended target groups of ICare interventions, an online questionnaire with potential facilitators/delivery staff and semi-structured interviews with policy makers.

**Conclusion:**

The challenge was to develop a design that allowed flexibility but at the same did not jeopardize the validity of the study. Implications drawn from this survey are not restricted to specific preventive interventions but will provide general information on how online mental illness prevention can be best implemented in various settings.

## Introduction

Research evidence for Internet-based interventions in the prevention of mental health problems across various target groups and conditions is increasing.^1–4^ However, the sustainable implementation and the dissemination across sectors and settings as well as the utilization of the research findings for health policy making remains a challenge. The implementation (defined as ‘a specified set of activities to put into practice an activity or program of known dimensions’^5^) and dissemination of new technologies requires change processes on the individual level (e.g. target group, professionals/staff), on the organizational level (e.g. healthcare organization)^6^ as well as on the system level (e.g. healthcare system). However, sectoral and organizational adoption, target group reach and implementation are influenced by a manifold of facilitating or hindering contextual factors, which are distinct for different sectors, settings and local conditions. Settings for health promotion and prevention are social contexts in which people pursue their daily activities, whereby individual, organizational and system behaviour are influenced by the interaction of individual, environmental and organizational factors.^7^ Therefore, each setting has its own specific processes and structures which have to be considered when implementing Internet-based prevention programmes for mental health problems.

For the healthcare sector, some knowledge on barriers and enablers for the uptake, implementation and maintenance of Internet-based interventions for mental health disorders is available.^8–10^ For instance, for eMental-Health (eMH) interventions for Mood Disorders a recent review found barriers related to (i) acceptance among patients, providers, organizations and healthcare settings, (ii) appropriateness of eMH to address the mental disorder, (iii) engagement in implementing and delivering eMH interventions, (iv) resources for implementing and delivering eMH interventions, (v) primary and facilitating processes in service delivery, (vi) leadership (directing and controlling working processes) and (vii) the healthcare system (policies, resources and collaborations).^10^ However, previous studies included predominantly Internet-based treatment. Knowledge about implementation barriers and facilitators of Internet-based prevention of mental health problems in real-world practice as well as for dissemination to the target groups in other settings, like schools and universities, is scarce. A recent systematic review on reach, adoption, implementation and maintenance of Internet-based interventions to prevent eating disorders in adolescents, which was also conducted in the course of the ICare project, showed that it is feasible to implement these interventions into the school setting and that a large number of adolescents can be reached via this setting. It yielded some knowledge on implementation facilitators and barriers; however, it revealed also a lack of large-scale dissemination studies, and a lack of reporting on external validity indicators relevant for the implementation and dissemination of these interventions in different settings and regions.^11^

The implementation of eMH interventions into routine practice can be improved by knowledge about settings and contextual factors as well as the involvement of relevant stakeholder groups. Moreover, a lack of broad context to evaluate external validity is a major impediment to dissemination of programmes. Thus, involving diverse stakeholders in the planning, implementation and evaluation processes of health promotion and prevention interventions is considered to increase their success.^8–10^ A detailed analysis of stakeholder interests and power relations helps to understand a particular setting.^12^ Besides the consideration of contextual factors and existing structures, the needs and characteristics of different stakeholder groups have to be taken into account as well. Stakeholder inclusion in eHealth research is especially important as it helps to foster sustainable cooperative contacts and learning processes.^13^ More importantly, the involvement of potential users or target groups in the design can help to create user-friendly programmes that have a higher likelihood of adoption and dissemination.^14–16^

Thus, in the first phase of ‘ICare’—a European research collaboration network with the overarching goal of establishing a comprehensive model of eMH service delivery—a stakeholder survey was developed to create knowledge necessary for successful implementation, dissemination and exploitation of ICare interventions in different settings.

ICare brings together evidence-based online interventions that span the mental health intervention spectrum including promotion of mental health, universal prevention, targeted prevention for vulnerable risk groups and self-help for people affected by recognisable symptoms of mental health conditions, for persons suffering from full syndrome eating disorders to bridge waiting time for treatment or for carers of people with an eating disorder.^17^ The interventions are implemented in three different settings (healthcare, university and school setting) across six European countries (Germany, Austria, Switzerland, UK, The Netherlands and Spain).

The aim of the ICare stakeholder survey was to explore—across different settings and stakeholder groups—stakeholders’ experiences with and their needs and attitudes regarding Internet-based preventive interventions of mental health problems (universal and targeted prevention) as well as possible hindering and fostering context parameters for their widespread and sustained implementation and dissemination to provide knowledge and guidance on the adaptation, evaluation and implementation of the ICare online preventive interventions in each country and setting.^18^ The aim of this article is to describe the development and the design of the ICare stakeholder survey, results of the stakeholder survey in the different settings (healthcare, university and school) are published in separate articles in this supplement.

## Methods

Since the aim of this stakeholder survey was to create knowledge across different settings and stakeholder groups, the process of developing an appropriate study design was crucial: first, key stakeholders needed to be identified, who are relevant across all interventions and settings of preventive ICare interventions. Based on these results, different methods for each stakeholder group needed to be determined following a mixed methods approach.

Since the Reach-Efficacy/Effectiveness–Adoption–Implementation–Maintenance (RE-AIM)^19^ model was applied as common evaluation framework in ICare, we used this framework as guidance in the development of the research questions of the stakeholder survey. However, the stakeholder survey did not refer to effectiveness/efficacy. Accordingly, the stakeholder survey addressed the following research questions: which (i) *experiences*, (ii) *needs*, (iii) *values* and *attitudes* do the different stakeholder groups have regarding online interventions to prevent mental health problems from being implemented into the existing healthcare systems and into specific settings like schools and universities? (iv) Which population groups are considered to be *underserved* regarding such interventions? (v) Which *hindering and fostering context factors* need to be considered to optimize *reach, adoption, implementation* and *maintenance* when implementing online interventions to prevent mental health problems into the existing healthcare systems and into school and university settings?

### Settings for the stakeholder survey—based on ICare interventions

The stakeholder survey design was based on the settings and target groups of the respective ICare interventions. In total, five ICare interventions involve universal or targeted Internet-based prevention programmes for a wide range of mental health problems including depression, anxiety, eating disorders and low resilience and two interventions focus on self-help programmes in the context of treatment for eating disorders. The interventions are designed to be implemented in the healthcare system (four interventions), schools (one intervention) and/or the university setting (three interventions) of participating countries. Each intervention is implemented in at least two countries. An overview of the ICare interventions as well as target groups, their related settings, partner countries and additional countries in which recruitment took place is depicted in Table 1.

**Table 1:** Overview of ICare interventions for the prevention and treatment of mental health problems

Intervention	Level of intervention^a^	Aim	Setting	Target group	Partner countries	Additional recruitment
‘Everybody’^20^	Universal and targeted prevention (tailored programme)	Promoting healthy body image and reducing risk for eating disorders	Healthcare System	Women ≥18 years	DE	AT, CH
‘Healthy Teens @ School’^21^	Universal and targeted prevention (tailored programme)	Promoting healthy lifestyle, stress coping, reducing risk for eating disorders and obesity	School setting	14- to 19-year-old students	AT, ES	
‘CORE’^22^	Targeted Prevention	Promoting resilience	University setting	University students with low resilience	ES, DE, CH	
‘ICare Prevent’^23^	Targeted Prevention	Reducing negative emotions like depression and anxiety	Healthcare System & University setting^b^	Individuals ≥18 with subclinical symptoms of depression or anxiety	DE, ES, CH, NL	AT
‘PLUS’^24^	Targeted Prevention	Reducing risk mental health disorder	University setting	University students at risk for mental health problems	UK, AT	IRL, DE
‘*everyBody* *Plus*’^25^	*Guided self-help in the context of treatment*	*Reducing and/or preventing binge eating*	*Healthcare System*	*Women ≥18 seeking outpatient treatment for Bulimia Nervosa or Binge Eating Disorder*	*DE, UK*	
‘*We* *Can*’^26^	*Guided self-help in the context of treatment*	*Increasing skills of carers to support individuals with Anorexia nervosa*	*Healthcare System*	*Carers of individuals with Anorexia nervosa*	*DE, UK*	

a Universal preventive interventions are usually targeted to the general public or a whole population that has not been identified on the basis of a risk for a specific disorder. Targeted prevention programs are targeted to individuals or a population subgroup whose risk of developing a specific disorder is significantly higher than average or to individuals who are identified as having signs or symptoms of a specific disorder but do not meet diagnostic levels at the current time. Guided self-help interventions in the context of treatment are targeted either to individuals who meet diagnostic levels at the current time and are on a waitlist for treatment to bridge waiting time for treatment or to carers of persons who are in treatment (parents, partners).

b ICare Prevent was implemented in the University setting in NL.

Abbreviations: DE, Germany; AT, Austria; CH, Switzerland; ES, Spain; NL, Netherlands; UK, United Kingdom; IRL, Ireland.

Since the majority of ICare online interventions are aimed at universal and targeted prevention of mental health problems (Table 1), the overall ICare stakeholder survey was developed for preventive interventions. For the self-help interventions implemented in the UK and in Germany, intervention-specific stakeholder surveys were conducted, because the stakeholder survey instruments had to be adapted accordingly. This article focuses on the study design of the overall stakeholder survey for prevention programmes. The respective designs along with the results for the self-help programmes can be found elsewhere in this Supplement.^27^^,^^28^

### Identification of key stakeholders

The following approach was applied to identify the most important external stakeholders, i.e.(groups of) people who are not directly linked to the ICare consortium but might have an interest in the programmes, might be affected by their implementation and/or can affect their uptake, implementation and dissemination. As a first step, we reviewed the literature on stakeholder surveys related to online interventions for the prevention and treatment of mental health disorders. At the time of designing the survey, no comprehensive survey was identified apart from a study exploring stakeholders’ views on digital treatment for depression conducted in the course of the E-COMPARED project.^29^ Informed by its stakeholder map, key stakeholder categories and potential representatives for the implementation settings of the ICare preventive interventions (Healthcare System, University Setting, School Setting) were identified and mapped by ICare consortium members from Austria. Finally, all members of the ICare consortium reviewed the proposed stakeholder groups, considering intervention and country specifics and agreed upon the final targeted stakeholder groups.

**Figure 1:**
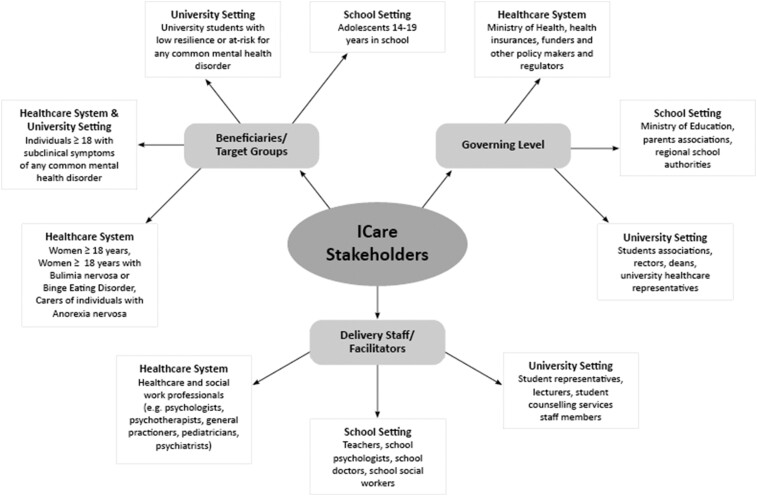
ICare stakeholder groups across different settings

We decided to approach three stakeholder groups which we considered to be most important: (i) *Target groups or beneficiaries* are the people or groups who are directly affected by, and for whom the individual programmes are designed. (ii) *Delivery Staff/Potential Facilitators* are individuals who directly work with the target groups, i.e. care for the beneficiaries or might offer the intervention to them and might be also directly affected in their everyday work, but on the other hand can also have a significant amount of influence on the implementation process and maintenance. Opinions from potential facilitators/delivery staff, such as teachers, university staff, psychologists, psychotherapists and physicians are especially helpful for gaining information on how these interventions can be best adopted by organizations and implemented in routine care. (iii) *Policy makers* are usually stakeholders at a governing level as well as expert consultants who are involved in the latest decision-making processes and have the power to contribute to the success or failure of the interventions as well as to decide on their further funding (e.g. representatives of insurances, ministries, etc.). They are able to provide information regarding important factors relevant for a long-term and sustainable implementation of Internet-based interventions. Details on the stakeholder groups approached are depicted in Figure 1.

Relevant representatives of the stakeholder categories in the individual countries were identified by ICare investigators who implemented preventive interventions in the respective country. The recruitment strategy for each stakeholder group and method is described in the respective section of this article.

### Mixed methods approach

Because each stakeholder group has unique characteristics, different survey methods were applied to each group, which resulted in a concurrent mixed methods design.^30^ We used quantitative as well as qualitative methods and aimed to synthesize the results and form meta-inferences at the end of the study: (i) *Focus groups*: In order to improve the individual programmes and to obtain valuable insights for their implementation and dissemination, focus groups with potential beneficiaries and target groups of the studies were planned. The group interaction in focus groups is mainly used to generate data and insights. Thus, guided responses move to a deeper and more considered level.^31^ This particular feature of focus groups is a crucial factor when conducting research with the actual target group of an intervention, since their ideas and attitudes are highly relevant for the design and the success of the intervention. (ii) *Online questionnaire:* To include a wide range of potential facilitators/delivery staff of the ICare interventions a confidential online questionnaire was developed. This method was chosen because an online questionnaire can easily be disseminated across a large number of stakeholders. (iii) *Semi-structured interviews:* Semi-structured in-person or telephone interviews with policy makers were planned because we assumed that individuals in high positions of ministries, insurances, healthcare providers and school/university authorities would be more likely to complete an interview than a questionnaire.

### Sampling and recruitment plan

Following a criterion based sampling strategy^31^^,^^32^ ICare partners were instructed to obtain stakeholder data for the overall stakeholder survey from the settings in which they planned to implement preventive ICare interventions (Table 1). For example, Germany and Switzerland planned to implement preventive interventions in the healthcare system and the university setting. Thus, stakeholder data needed to be obtained in those two settings. UK planned to implement a preventive intervention in the university setting only, thus data for the overall stakeholder survey was solely obtained in this setting.

To determine the planned number of focus groups and semi-structured interviews we considered the concept of data saturation^31^ (i.e. a point when expanding the number of focus groups or interviews would only produce repetitive information) in a way to best meet our research objectives and by drawing on experts’ recommendations and standards for the field (i.e. 15–30 interviews per project;^33^ no more than 50 interviews in total^31^ and a minimum of two focus groups^34^) Besides, we aimed to achieve saturation within each interview and focus group by thoroughly probing the interviewees and by respective written interviewer instructions. The sample size for the online questionnaire was determined by *a priori* power analysis (section *Online questionnaire with potential facilitators/delivery staff*). An overview of the planned number of focus groups, semi-structured interviews and planned sample size for the online questionnaire is depicted in Table 2, and the sampling and recruitment strategies for the different methods are described in detail in the following sections. Incentives were determined individually by each country, depending on their local circumstances. Details on the particular recruitment strategies in different settings and countries can be found in the respective papers in this Supplement.^35–37^

**Table 2:** Planned number of focus groups, semi-structured interviews and online survey participants per setting and country

	AT	CH	DE	ES	NL	UK	Total
**Focus groups with target groups (Number of planned focus groups)a**							
Healthcare system							10
“Everybody”			2				
“ICare-Prevent”	2	2	2	2			
University setting							12
“CORE”		2	2	2			
“PLUS”	2					2	
“ICare-Prevent”					2		
School setting	2			2			4
“Healthy Teens @ School”							
**Total**	**6**	**4**	**6**	**6**	**2**	**2**	**26**
**Semi-structured interviews with stakeholders on governing level (Number of planned interviews)**							
Healthcare system	4	4	4	4			16
University setting	4	4	4	4	4	4	24
School setting	4			4			8
**Total**	**12**	**8**	**8**	**12**	**4**	**4**	**48**
**Online questionnaire for delivery staff (Planned sample size)**							
Healthcare system	20–30	20–30	20–30	20–30			80–120
University setting	20–30	20–30	20–30	20–30	20–30	20–30	120–180
School setting	20–30			20–30			40–60
**Total**	**60**–**90**	**40**–**60**	**40**–**60**	**60**–**90**	**20**–**30**	**20**–**30**	**240**–**360**

a Participants for the focus groups were recruited based the inclusion criteria of ICare interventions (Table 1).

DE, Germany; AT, Austria; CH, Switzerland; ES, Spain; NL, Netherlands; UK, United Kingdom.

#### Focus groups with target groups and beneficiaries

The sampling strategy for the focus groups was based on the individual intervention characteristics since different risk groups had to be considered (Table 1). A minimum of two focus groups^34^ per intervention and participating country was planned, resulting in 26 focus groups. Ideally, participants should be recruited based on the focus of the intervention, such as social demographics and the inclusion and exclusion criteria of the individual studies.^20–24^ The guideline for the ICare partners was to recruit between five and eight participants per focus group.

#### Online questionnaire with potential facilitators/delivery staff

Although only exploratory analyses of group differences were planned, we conducted an *a priori* power analysis to plan the sample size for the online questionnaire. The analysis was based on the intention that at least a medium effect size (*f* = 0.25) for differences between settings in the level of stakeholders’ preference to implement them as primary outcome could be detected by a statistical test and was determined by using G*Power^38^ based on the assumption of ANOVA (Alpha 5%, Power 80%). This resulted in at least 20–30 datasets per country and setting and led to a total sample size across all settings and countries of 240–360 participants. Consortium partners were instructed to send the link of the final version of the online questionnaire via e-mail to representatives of the three involved settings: for example, a random sample of general practitioners, psychologists, psychotherapists and so on (healthcare system) and students’ associations/groups, student representatives, university/school psychological services, university/school counselling services and university/school teachers (school and university setting). ICare partners were asked to recruit participants via different channels for each setting to get as close as possible to a representative sample.

#### Semi-structured interviews with policy makers

Depending on their availability and time we interviewed policy makers in-person or via telephone. Following a criterion-based sampling strategy, we aimed to conduct four interviews per country and setting, which resulted in a minimum of 48 interviews in total. This is in accordance with general literature recommendations stating that qualitative interview samples for a single study often lie under 50.^31^ ICare partners were asked to identify relevant representatives of this stakeholder group in their countries. No further standards were set. However, ICare partners were instructed to collect background information on different interviewee characteristics, such as organization type, number of years of experience in the respective sector, level of influence/power (e.g. members of authority organizations and ministries are considered to have high influence, [social] insurers moderate influence and researchers/consultants low influence).

### Development of instruments

The topic guides for the focus groups and interviews as well as the questions of the online questionnaire were strongly oriented towards the research questions informed by the RE-AIM framework.^19^ Thus, the topics addressed stakeholders’ (i) experiences with Internet-based interventions to prevent mental health problems and disorders, (ii) needs regarding such interventions including topics, characteristics and aims, (iii) values and attitudes regarding such interventions, (iv) underserved groups which might benefit most from Internet-based preventive interventions in the field of mental health from stakeholders’ point of view, as well as (v) hindering and fostering context factors for the dimensions reach, adoption, implementation and maintenance, which need to be considered when implementing online interventions in the respective settings (based on the RE-AIM framework). Consequently, a definition of each RE-AIM dimension was provided followed by questions about hindering and fostering factors for each dimension regarding the particular setting. For example, ‘REACH is defined as the absolute number, proportion and representativeness of individuals who are willing to participate in a given initiative’. Accordingly, we asked policy makers ‘Which factors could foster REACH of pupils/university students/individuals regarding the use of an Internet-based intervention program that aims to prevent mental health problems and disorders?’. Additionally, the development of the instruments was informed by the E-COMPARED online survey which was used to explore stakeholders’ views on digital treatment for depression.^29^ We reviewed the questions used in the E-COMPARED questionnaire regarding their applicability for inclusion in the ICare stakeholder survey and adapted those applicable according to our research questions. For example, we derived questions from the ‘attitudes’ survey theme of the E-COMPARED questionnaire^29^ such as advantages and disadvantages of Internet-based interventions compared to face-to-face interventions as well as weighing the advantages and disadvantages, and the level of support of stakeholders for Internet-based prevention of mental health problems in the school/university/healthcare setting. ICare consortium members from Austria developed initial English versions of all instruments, which were reviewed by other consortium members. Based on the revised English versions ICare consortium members from Austria, Spain, Switzerland and The Netherlands translated the survey instruments into German, Spanish and Dutch, respectively. A forward-backward translation was applied for the German and English versions. Spanish and Dutch researchers checked back with the team when issues regarding lingual interpretation arose.

In order to assure comparability across all methods, all questions and items used in the topic guides for the focus groups and semi-structured interviews and online questionnaire were designed accordingly. The instruments were designed to be adaptable to the healthcare system, school or university setting, depending on which setting the participant(s) or respondent(s) belonged to. Instructions on how to conduct, transcribe and analyse interviews and focus groups and a template regarding recruitment and facilitation processes were sent out to all partners to ensure consistency across countries and settings. The online questionnaire was created with LimeSurvey (http://www.limesurvey.org), an open source survey application and consisted of 27 items (with several sub-items) including closed-ended and open-ended questions. The questionnaire was pilot-tested in Austria by applying the ‘think-aloud’ technique with four persons representing the different settings, resulting in wording and format changes.

The stakeholder survey instruments (topic guides for the focus groups and semi-structured interviews and the online questionnaire) are provided in Supplementary document S1.

### Data analysis plan

Since a mixed methods design was applied, we integrated quantitative and qualitative approaches to the data in the analysis and interpretation stage of the study.^30^

The data of the online questionnaire from Limesurvey were extracted and analyses were performed in IBM SPSS statistics 22.0 and ©Microsoft Excel 2010. Descriptive statistics were calculated for each closed-ended question and differences between countries and settings were analysed by statistical tests appropriate to the scale level and distribution of variables. To analyse differences between settings and countries, Chi-square tests were used for categorical variables and ANOVA or Kruskal–Wallis tests were used for continuous variables depending on the distribution of variables. Alpha was set to 5%.

The focus groups and interviews were transcribed verbatim in German, English, Dutch and Spanish language. Dutch and Spanish transcripts as well as Dutch and Spanish answers to open-ended questions in the online questionnaire were translated by researchers in The Netherlands, Spain and Austria into German or English language and a thematic analysis approach was applied.^39^ The transcripts and the open-ended questions of the online questionnaires were coded and organized in NVivo 11 Pro software. The primary objective of the overarching analysis was to identify a wide range of relevant themes rather than providing quantified measures. Two researchers coded and crosschecked the transcripts independently by identifying themes based on the research questions as well as emerging themes and their relevant subcategories. Subcategories were structured and compared, and a categorical network was produced. Independently from the focus groups and interview transcripts, the same procedure was applied to the open answer categories of the online questionnaire. Finally, the results were merged. Several members of the research team interpreted the final categorical network.

### Data protection and ethical issues

The stakeholder survey was conducted in accordance with the ethical standards outlined in the 1964 Declaration of Helsinki and its later amendments. Ethics approvals were obtained from the relevant local ethics commissions of all consortium partners participating in the stakeholder survey. Informed consents including consents for audio recordings were obtained from all stakeholders participating in the focus groups and interviews. Additionally, for participants younger than 18 years (school setting), we obtained informed consent from legal representatives. The completed informed consent forms that contain full names of participants were stored offline only. Audio files were stored on a secured and password protected server at the Ferdinand Porsche FernFH—Distance-Learning University of Applied Sciences (Austria). Information that allows the potential identification of participants (e.g. names, name of organizations) was not transcribed. In the online questionnaire, data was obtained anonymously. No personal data like name or e-mail address was needed to access the online questionnaire. Data was transmitted via a secured connection and stored on a secured and password protected server at the Ferdinand Porsche FernFH—Distance-Learning University of Applied Sciences (Austria).

## Discussion

While there is increasing evidence on the efficacy of Internet-based prevention of mental health disorders and promotion of mental health the sustainable implementation into routine practice across different settings remains a challenge. An important prerequisite for the sustainable implementation, which may not have received sufficient attention in previous studies, is the consideration of the specific structures and processes of the implementation setting^6^^,^^7^ as well as local context factors. Involving multiple stakeholders in the planning and implementation can help to understand a particular setting and is considered to increase the success of an intervention. Accordingly, this stakeholder survey aimed to reveal valuable insights into the experiences, needs, values and attitudes towards Internet-based programmes for the prevention of mental health problems, factors relevant for reach, adoption, implementation and maintenance of such programmes as well as knowledge on which population groups are considered as underserved in the healthcare, school and university setting across six European countries. Different perspectives were considered by including three key stakeholder groups: target groups/beneficiaries, delivery staff/facilitators and policy makers.

There are clear challenges in designing a study that includes multiple target groups, perspectives, settings and countries. In order to gather rich information regarding the same overarching research questions on the one hand and to meet the requirements resulting from the unique characteristics of the different stakeholder groups on the other hand, there is no ‘one size fits all’ approach. At the same time, the study design needs to be flexible to such an extent that researchers are allowed to quickly respond to local circumstances and contextual factors, and yet not jeopardize the validity of the study. Even if all members of the research team use the same instruments and adhere closely to the procedure, due to characteristics of the different target groups and local circumstances, differences across countries may occur. Thus, members of the research team across countries and interventions need to take this into account throughout data analysis and interpretation processes by particularly paying attention to possible local study limitations.

Conclusions drawn from this survey are not restricted to interventions targeting specific mental health conditions or specific types of interventions. We included a wide range of mental health problems and covered the whole spectrum of preventive interventions from health promotion and universal prevention to selected and indicated prevention. The results will show how interventions included in the ICare project can be improved as they are implemented and will inform the development, implementation and dissemination of future Internet-based interventions in this field. Ideally, the survey results will also shed light on how existing interventions can be scaled up so that more individuals can benefit from technology-based approaches in mental health promotion. In this context, this study also contributes to the WHO’s mental health action plan, which proposes strengthened evidence and research, the implementation of strategies for mental health promotion and prevention, the integration of mental health services in community-based settings and effective leadership and governance for mental health.^40^

## Supplementary data

Supplementary data are available at *EURPUB* online

## Supplementary Material

ckab045_Supplementary_Material
